# Would the control of invasive alien plants reduce malaria transmission? A review

**DOI:** 10.1186/s13071-018-2644-8

**Published:** 2018-02-01

**Authors:** Christopher M. Stone, Arne B.R. Witt, Guillermo Cabrera Walsh, Woodbridge A. Foster, Sean T. Murphy

**Affiliations:** 1 Illinois Natural History Survey, University of Illinois, Urbana, Champaign, IL 61820 USA; 2 CABI Africa, 673 Limuru Road, Muthaiga, PO Box 633-00621, Nairobi, Kenya; 3 Fundación para el Estudio de Especies Invasivas (FuEDEI), Bolivar 1559, Hurlingham, Buenos Aires, Argentina; 4 Department of Evolution, Ecology and Organismal Biology, Ohio State University, Columbus, OH 43210 USA; 5 CABI, Bakeham Lane, Egham, Surrey, TW20 9TY UK

**Keywords:** Invasive alien plants, Biological control, Environmental management, Plant-vector interactions, Nectar feeding, Resting, Larval habitat, Vector-borne disease

## Abstract

Vector control has been the most effective preventive measure against malaria and other vector-borne diseases. However, due to concerns such as insecticide resistance and budget shortfalls, an integrated control approach will be required to ensure sustainable, long-term effectiveness. An integrated management strategy should entail some aspects of environmental management, relying on coordination between various scientific disciplines. Here, we review one such environmental control tactic: invasive alien plant management. This covers salient plant-mosquito interactions for both terrestrial and aquatic invasive plants and how these affect a vector’s ability to transmit malaria. Invasive plants tend to have longer flowering durations, more vigorous growth, and their spread can result in an increase in biomass, particularly in areas where previously little vegetation existed. Some invasive alien plants provide shelter or resting sites for adult mosquitoes and are also attractive nectar-producing hosts, enhancing their vectorial capacity. We conclude that these plants may increase malaria transmission rates in certain environments, though many questions still need to be answered, to determine how often this conclusion holds. However, in the case of aquatic invasive plants, available evidence suggests that the management of these plants would contribute to malaria control. We also examine and review the opportunities for large-scale invasive alien plant management, including options for biological control. Finally, we highlight the research priorities that must be addressed in order to ensure that integrated vector and invasive alien plant management operate in a synergistic fashion.

## Background

Malaria remains a major threat to human health in many parts of the tropical and sub-tropical regions of the world [1], even though significant progress has been made with the implementation of preventative measures such as insecticide-treated nets [2, 3]. In sub-Saharan Africa for example, the disease remains chronic in many countries, and while mortality rates have fallen, an estimated 855,000 deaths were caused by this disease in 2013 [4]. Although the use of insecticide-treated nets has significantly reduced infection rates, there is concern about the increase in insecticide resistance in *Anopheles* spp. (Diptera: Culicidae), the vectors of *Plasmodium* spp. [5]. For instance, various pyrethroid resistance mechanisms are now common throughout much of the western and central parts of Africa [6]. In the past, pesticide use in agro-ecosystems also had a significant impact on malaria vectors, and likely has selected for insecticide resistance [7–9]. At the same time the natural enemies of mosquitoes continue to be negatively affected through the use of agro-chemicals [10]. As such, there is an increased risk that gains in malaria control will be negated, unless an integrated and sustainable approach is developed and implemented.

An integrated approach to vector control would manage factors contributing to mosquito reproduction, and longevity, in a sustainable and efficacious manner [11, 12]. Such strategies will rely on collaboration and coordination between multiple disciplines and societal stakeholders. Important cross-disciplinary research questions are those relating to (i) coordinating agricultural and vector control practices, given the selective pressures for resistance traits; (ii) understanding how changes in vegetation structure and composition (whether due to deforestation, to land-use changes, or to the spread of invasive plants - the last, the focus of this review) will affect pathogen transmission; and (iii) understanding the specific effect of plants on mosquito survival, biting frequency, reproduction, and vector competence. Emerging evidence from several sources indicates that many invasive alien plant (IAP) species may be particularly important in the enhancement of mosquito demographic parameters [13, 14].

Management of IAP species and control of vector-borne diseases such as malaria typically are not considered simultaneously. Yet, if there are sufficiently strong interactions between invasive plants and vectors, vector-control activities targeted at one may impact the other, directly or indirectly. If such interactions are negative, policy makers will have to weigh the repercussions at the level of the environment, economic development and human health. If the interactions are positive, there may be exploitable synergies between control of invasive plant species and vectors. In this review we explore whether there are reasons to expect such interactions and review the literature on the topic. We focus on malaria, though resulting insights will likely apply more broadly and beyond mosquito-vectored pathogens. For instance, an accumulating body of evidence suggests that IAPs can affect the spread and transmission intensity of sleeping sickness as well as various tick-borne pathogens [15–18].

IAP species are now a major problem in Africa and other regions where they have significant negative impacts on crop and pasture production, human and animal health, water, and other natural resources [19–25]. These plants were introduced over the course of the last few centuries, either accidentally or deliberately, via increasing trade and transport. Most are now widely distributed and still spreading, a situation which is exacerbated by increasing disturbance, land transformation [26, 27] and climate change [28, 29].

Given their vast distributions, there are at least two important ways that IAP species may be significantly influencing the biology and malaria-transmitting ability of *Anopheles* spp. First, female and male mosquitoes need sugar sources for energy, mostly obtained from floral and extra-floral nectar, honeydew and fruits [30–32]. Secondly, many IAPs provide suitable habitats as resting or breeding sites for mosquitoes [33, 34]. An open question is whether and how these aspects of mosquito biology are different in environments dominated by invasive plants, and whether this has ramifications for the transmission intensity of malaria. The eventual aims of the research must be to confirm that *Anopheles* spp. are, in reality, more abundant, with better reproduction, survival, and other vectorial-capacity characteristics in the presence of IAPs and to confirm that malaria incidence is higher where there is an abundance of non-native plants. Present evidence indicates that this is true [35], but our knowledge of *Anopheles* plant hosts is meagre, and native plant species also are known to provide malaria vectors with benefits. The inferred links between *Anopheles* spp. and IAP species raises the associated question of whether the management of particular invasive plants would contribute to the suppression of *Anopheles* spp. populations and to a reduction in malaria incidence.

Currently, much of the relevant literature on these topics is scattered. Thus, the purpose here is to provide a review of the current state of knowledge about these topics and to identify the gaps in this knowledge. The review is divided into three major sections. The first covers the questions of whether *Anopheles* spp. benefit from IAPs and, if so, whether this has a positive influence on the rate of malaria transmission. We then turn to the questions of the potential for management of IAPs on a large scale; and finally, discuss whether invasive plant control is likely to result in a reduction in the incidence of malaria. Although the main theme of this review is the relation *Anopheles* mosquitoes have with IAPs, we have included studies of other genera of mosquitoes that contribute to a general understanding of the topics.

## Do invasive alien plants have a positive influence on the rate of malaria transmission?

### Measures of transmission rate: The basic reproduction number and vectorial capacity

Here, we focus on how and whether the presence of individual or various functional groups of invasive plant species may affect the rate of *Plasmodium falciparum* malaria transmission. An intuitive way to explore this is to focus on the basic reproduction number of malaria, *R*_0_, its constituent parameters, and how those parameters are affected by functional traits or ecosystem impacts typically associated with invasive plant species.

The basic reproduction number provides an estimate of the number of new infections in humans following the introduction of a single infected case into a fully susceptible population [36]. It is described by the following eq. [37]:

1 $$ {R}_0=\frac{ma^2{bce}^{-\mu \tau}}{\gamma \mu} $$

This can be understood as the product of the number of mosquito bites per person per day (*ma*), the duration a typical human remains infective ($\frac{1}{\gamma }$), the probability of a mosquito becoming infected upon biting (*c*), the probability of a mosquito surviving the extrinsic incubation period (*e*^−*μτ*^), the number of bites per mosquito over its expected lifespan ($\frac{a}{\mu }$), and the probability that an infectious bite will establish an infection in a human (*b*). Certain of these vector-related properties can be broken down further. For instance the biting rate of mosquitoes on humans, *a*, can be seen as the product of the inverse of the average time between blood meals and the preference to bite humans over other types of animals, such as cattle [37].

Vectorial capacity is a measure strongly related to the basic reproduction number. It was initially defined by Garrett-Jones [38] and isolates the entomological parameters from *R*_0_. Vectorial capacity, *C*, relates to *R*_0_ as${R}_0=\frac{b}{\gamma }C$ [37]. It represents the number of potentially infective bites that would result from an infected human being exposed to a mosquito population for a single day, and it provides a useful lens through which to explore the link between vector traits, IAPs, and malaria transmission.

All these vector traits, including complications such as senescence or traits that can lead to heterogeneous exposure, such as biting preferences or vagility, may be affected by plant-species composition and abundance in a given area. Evidence for this as it relates to nectar-feeding by mosquitoes has recently been reviewed [39]. Furthermore, theoretical calculations of the impact of attractive toxic sugar baits for malaria control demonstrate that the availability and distribution of plant-nectar sources are significant determinants of malaria inoculation rates [40]. Here, we summarize these plant-vector interactions briefly and highlight other potential effects of plants on vectors, particularly those associated with IAPs.

### The relationship of mosquitoes and plants

It has been known for over a century that adult mosquitoes are, in part, phytophages that feed on sugar sources from nectar and other plant juices [30, 41, 42]. Females need blood mostly to mature their eggs [43], although some nourishment is obtained from blood itself in most species [44]. Blood meals can come from a wide variety of hosts, perhaps even other insects, so mosquitoes are eminently plastic host feeders [45, 46]. The few instances of stenophagy, as is the case of *Anopheles gambiae* (*s.s.*) on humans, is also particularly relevant, because this specificity is part of its great efficiency as a malaria vector [45]. Although the females of some mosquito species seem to be blood specialists [47], even these females still feed on sugar during certain age and reproductive stages or under conditions of host- or oviposition-site scarcity [48–50].

Many different plant species are fed on, but not all plants have the same attractiveness, or effect on survival and flight [31, 32, 51–53]. Yet laboratory experiments on *An. gambiae* [54] indicated that any plant was better than no plants, even *Lantana camara* L. (Verbenaceae), a species reported to have mosquito-repellent properties [55]. Furthermore, plant choice can show seasonal, diel, and even sexual differences [32]. The extent and consequences of plant dependence on the ecology of adult mosquitoes and their vectorial capacities is a field of active research.

A currently less explored relationship between mosquitoes and plants is that between larvae and aquatic plants. Mosquitoes have aquatic larvae, and a great many different kinds of interactions can be expected between larvae, ovipositing adults, and aquatic plants. This issue was first approached in the Americas in the early twentieth century in relation to malaria outbreaks in the southeast USA and the Panama Canal area [56–58]. In those days it was considered that malaria control would be achieved essentially through larval control. In fact, the only instances in history of successful mosquito eradication (*Aedes* and *Anopheles* spp.) were achieved mainly by targeting larvae and breeding site management, as in the cases of *An. arabiensis* eradication in Brazil and Egypt between the 1930s and 1940s [59–61]. But since the appearance of DDT in the 1940s, mosquito control has concentrated on adult control, and as a result many knowledge gaps on larval ecology of mosquitoes persist [62]. However, concerns about insecticide resistance, environmental impacts, rising costs of indoor spraying, and logistical constraints, have sparked renewed interest in larval control of malaria vectors [60, 62].

### Nectar-plant contribution to transmission pressure

For most of the traits that determine the vectorial capacity of malaria mosquitoes, there is ample evidence that they are affected by plants. The effect of access to different plant species and their nectar on mosquito survivorship has been studied in detail. For instance, while access to nectar consistently increases longevity, there are large differences between different plant species with regard to their effect on longevity [50, 51, 54, 63, 64]. Besides the effect of nectar quality, abundance, and accessibility, some plants also provide mosquitoes with shelter: certain plants may create more suitable microclimates facilitating mosquito resting behaviour and diurnal survival. The extent to which this contributes to mosquito survivorship may depend on the mosquito species in question or the environment (e.g. the need or tendency to rest outdoors). The importance of such harbourage is not well-known, and resting behaviour in general remains an understudied aspect of mosquito biology.

The effects of plant nectars on the biting frequency of mosquitoes are less clear. In certain experiments, the biting rate is decreased when mosquitoes have ad libitum access to sugar sources [50, 65, 66]. In other cases, the biting rate is unaffected [67] or greater [64] with access to nectar-bearing plants or plants that are more attractive and/or produce more copious amounts of nectar. This discrepancy could be due to differences in host availability. For instance, mosquitoes are more likely to feed on nectar, and take larger meals, when blood hosts are available for only a short period of the night or at times that do not coincide with the species’ peak biting activity [68]. This would suggest that under natural field conditions where blood hosts are abundant and easily accessible, plants may have little impact on the biting frequency but affect other aspects of vectorial capacity and reproduction [67].

The development of *Plasmodium* within the mosquito may also be affected by feeding on different plant species. This development includes both the probability of the pathogen reaching the infective sporozoite stage (i.e. vector competence) and the average length of time between ingestion of a gametocytemic blood meal and appearance of sporozoites in the salivary glands (i.e. the extrinsic incubation period of *Plasmodium*). Besides sugars, nectar contains amino acids and secondary metabolites. These various compounds, either directly or indirectly (e.g. by affecting the mosquito’s immune response), may have an impact on pathogens within the vector. For instance, in *An. coluzzii*, females that had access to the fruit *Lannea acida* A. Rich. (Anacardiaceae) or the flowering ornamental plant *Barleria lupulina* Lindl. (Acanthaceae) were more likely to have disseminated sporozoites in their heads and thoraxes after an infectious meal than females that were exposed to a different ornamental, *Thevetia neriifolia* Juss. ex Steud. (Apocynaceae) [69].

The impact of plant-nectar on mosquito population density is complex, as numerous traits will affect this outcome. These traits will include female fecundity and survival. Studies that have measured the impact of sugar on the net reproduction rate of mosquito populations indicate a depressing effect of sugar [39]. However, because fecundity in mosquitoes is strongly correlated with the biting rate, this may not hold under field conditions (e.g. if differences in fecundity are merely a result of differences in the biting frequency). Another complication is that the regulation of mosquito population density, particularly of *Anopheles* spp., under natural conditions remains poorly understood. While seasonal changes in rainfall are clearly an important driver of population densities, density-dependent larval development may be relevant for part of the year or in certain larval habitats. If larval habitats are at carrying capacity during mid- to late rainy season, differences in population growth rates may not result in different population sizes.

The growth rate of a population can also be influenced through effects of plants on male mosquitoes. For male mosquitoes, feeding on nectar provides their sole source of nutrients. Without nectar, their prospects of survival and mating dwindle. Laboratory cage and mesocosm experiments indicate that in the absence of nectar, insufficient females may become inseminated to sustain a population [70, 71]. Whether this is applicable under natural conditions, where nectar sources may vary in attributes such as quality, quantity and accessibility, but likely are not entirely absent save for the most inhospitable environments, is not yet resolved. Females may not become inseminated until a later age in areas where sugar is inaccessible [67], and presumably such a delay in female reproduction affects the population growth potential.

### Plants and mosquito oviposition and larval development

The effect of aquatic plants on mosquito oviposition and larval development has received the most attention. Most studies indicate that some aquatic plants, both macrophytes and charophytes, boost mosquito reproduction or larval survival, while others inhibit it, often in contradictory reports [72]. The evidence suggests that aquatic plants contribute to the spread of many human diseases around the world, including malaria [14], although not all vectors respond the same way to them. The general description of breeding sites for *An. gambiae* (*s.l.*) are small pools or puddles with diameters less than 1 m, while vegetation is considered crucial for the breeding of *An. funestus* (*s.l.*) [73–77]. For instance, in the Lake Victoria region in East Africa, the most aggressive *Anopheles* spp. were not abundant in deep permanent lake waters, even if invaded by the IAP *Eichhornia crassipes* (Mart.) Solms (water hyacinth, Pontederiaceae) and other aquatic weeds. However, they bred abundantly in temporary or seasonal aquatic coastal habitats such as pools and swamps, more so when infested by aquatic vegetation [78]. Although experimental evidence is scarce, there are plenty of observational reports of a relationship between larval abundance and aquatic vegetation. Water lettuce, *Pistia stratiotes* L. (Araceae), the main host of *Mansonia* spp. mosquitoes, was also reported as a plant that favoured anopheline establishment [79, 80]. So were water primroses, *Ludwigia* spp. (Onagraceae) [81], another genus with several species that are invasive in many countries. The South American invasive plants *E. crassipes, Egeria densa* Planchon (Brazilian elodea; Hydrocharitaceae), *Hydrocotyle ranunculoides* L. (floating pennywort; Araliaceae), and water lettuce, were deemed to increase the risk of the return of malaria in Europe [82]. Water hyacinth and *Myriophyllum aquaticum* (Vell.) Verdc. (parrots feather; Haloragaceae) were also reported to stimulate anopheline reproduction in the USA [72, 81, 83]. Curry [57] and Rozeboom [58] stated that submerged species in the genera *Chara* (Characeae), *Utricularia* (Lentibulariaceae) and *Najas* (Hydrocharitaceae) provided vast breeding sites for *An. albimanus*, the most important malaria vector in the Americas. Studies in India state that aquatic plants, especially water hyacinth, were associated with increased *Anopheles* spp. richness, though not abundance [84]. In fact, aquatic weed control is a standard procedure in mosquito management [62].

Field studies using systematic samplings and statistical analyses (PCA and other multivariate analyses) corroborate the important role of aquatic plants in mosquito abundance. Rejmánková et al. [85] predicted biting risk for *An. albimanus* based on wetland characteristics with a 90 to 100% precision. These characteristics were emergent macrophytes, water hyacinth, dense cyanobacterial mats, and distance to the nearest human community. Subsequent studies by Rejmánková et al. [86, 87] confirmed that submersed and floating plants, and cyanobacterial mats were associated with *Anopheles* spp. abundance in Belize and Mexico. In Argentina, *Anopheles* spp. abundance, as well as that of several other species, was associated with the emergent/floating macrophytes *H. ranunculoides*, *Alternanthera filoxeroides* Grisebach (alligator weed; Amaranthaceae) and *Ludwigia* spp., as well as water lettuce and the floating ferns *Salvinia* spp. (Salviniaceae), all of which are important IAPs in many parts of the world. The chemical properties of the water courses sampled were not a relevant factor in the statistical analyses [88, 89]. Similar results were obtained in Mexico, where the dominant factor was water hyacinth during the dry season, and emergent Cyperaceae during the wet season [90]. Sinka and collaborators [75, 76, 91] published three extensive spatial analyses and reviews of the available knowledge on occurrence, distribution, and ecological requirements for the 41 main malaria vectors in the world. The data they obtained from their meta-analyses showed that some *Anopheles* spp., including the *An. gambiae* complex, are typically found in small water bodies devoid of plants, but most species are associated with aquatic plants and plant debris. Yet, even the *An. gambiae* species are often found in abundance in plant dominated waters. This was attributed to the typical adaptability of mosquitoes and to differences in behaviour at population level.

*Azolla* (Azollaceae) and *Salvinia* (Salviniaceae), two genera of floating ferns, and duckweeds (Araceae), have been reported both to prevent and favour mosquito establishment [92–94]. *Salvinia* spp. were proposed as a mosquito control option, acting apparently by simply providing a physical barrier to ovipositing females [93]. In the case of *Azolla* spp., the plant was described as trapping emerging adults in the multi-layered and multi-dissected fronds [95]. On a closer look, however, it would seem that the main factor is not so much plant species, but density [86, 96]. Pool experiments and systematic sampling of rice fields suggest that duckweed and aquatic ferns may hinder mosquito growth at high densities, but stimulate it at low densities [97, 98], and more than 80% cover by *Azolla* spp. must be kept, to provide mosquito control in rice paddies [98, 99]. Yet, the highest densities of fish that serve as mosquito predators occur at intermediate plant densities. Both very high and very low plant densities in lakes were associated with seven-fold reductions in fish densities [100]. Thus, despite the vector-suppressing aspects of aquatic vegetation, managing their densities at optimum levels may be unrealistic. Furthermore, severe invasive plant infestations often contribute to other problems, such as the loss of aquatic biodiversity, fouling of water resources, and the promotion of snail populations that serve as intermediate hosts of the blood fluke *Schistosoma* [101, 102].

There have been a few experimental approaches to understanding the relationship between mosquito breeding and aquatic plants. Furlow & Hays [97] planted pools with different aquatic plants, with a clean pool as a control, and allowed spontaneous mosquito colonization. The clean pools, and those with submerged macrophytes produced more *Anopheles* spp. than those with a thick duckweed cover. Orr & Resh [103] evaluated experimentally the relationship between plant cover and predation of mosquito larvae. Experiments whereby algae were mechanically extracted from natural streams demonstrated that water devoid of filamentous algae harboured dramatically fewer *Anopheles* larvae [104, 105]. The experimental and observational evidence indicates that the way aquatic plants benefit mosquito larval survival is by protection from predators [83, 103, 104, 106, 107], increased food availability [83, 104, 108], and dampened wind action [77, 108–110]. It is evident, then, that large, open, wind- and predator-exposed waters are deleterious to *Anopheles* spp. larvae in general.

Both terrestrial and aquatic vegetation affect immature mosquito dynamics in a number of ways. One way is by influencing where mosquitoes lay their eggs, which could affect local population densities. For instance, *Aedes albopictus* females appear to have a preference to oviposit in sites adjacent to flowering plants (*Buddleja davidii* Franch; Scrophulareaceae) [111]. Likewise, up to a certain density of *Myriophyllum aquaticum* cover, the oviposition activity of *Anopheles hermsi* was increased [83]. In choice tests, *An. gambiae* is more likely to oviposit on bare soil than on water near grassy vegetation [112], while *An. minimus* (*s.l.*) prefers to oviposit on water near small-leaved plants [113]. In The Gambia, presence of anopheline larvae was positively associated with short emergent vegetation or tufts of grass, but was lower in sites where taller vegetation shaded more than 25% of a potential larval site [114]. The latter insight is well established for certain sun-loving vectors and has led to the planting of species providing dense shade to control vectors. For instance, in Java, *An. maculatus* populations on tea plantations were managed by planting the invasive alien shrub *Tithonia diversifolia* (Hemsl.) A. Gray (Asteraceae) [115].

Terrestrial plants also can impact larval development through input of plant materials into water bodies. A well-known example is the deposition of maize pollen into larval habitats. In the vicinity of maize fields, larvae develop into adults with greater probability, do so more rapidly, and produce larger-bodied adults [116]. Larvae also were found to develop into adults despite conditions of intense crowding, if they were near areas where maize pollen was abundant [117]. The importance of the input of plant material in the form of leaf litter, fruits, or flowers is well established for the larvae of container- or treehole-breeding mosquitoes [13]. Additionally, certain IAPs can serve as harbourages for adults (e.g. Amur honeysuckle, *Lonicera maackii* Rupr; Caprifoliaceae) and support greater mosquito densities [118, 119]. The importance of such inputs for malaria mosquitoes in particular is less clear.

### Do mosquito interactions with invasive alien plants cause differences in transmission?

Does malaria transmission pressure differ between pristine and invaded landscapes? There are at least two ways by which vector-borne pathogen transmission might increase when invasive plants become established. The first would occur where an IAP establishes itself in a barren or early successional environment, or reaches a greater biomass (and therefore provides more adult refugia and nectar) than the plant species it replaced. The second scenario would occur when an IAP possesses traits that change the functioning of an ecosystem in a manner that enhances a vector’s vectorial capacity by altering its components.

A wide variety of ecological and evolutionary hypotheses have been posited as potential explanations for what makes particular species successful invaders, many of which allude to particular traits that allow for invasiveness [120–124]. These include the ideal-weed hypothesis, which suggests that a weed possessing life history traits such as early and high fertility, small seed size, and rapid growth would tend to have a competitive advantage. IAPs may have an advantage due to biotic release from enemies (enemy release hypothesis), whereby herbivores or pathogens that limit population growth in the native habitat are absent in invaded habitats. This would potentially allow for resources initially allocated to defences to be diverted to increased fertility or growth (evolution of increased competitive ability) [125–127]. Alternatively, invasive plants may release allelopathic chemicals to which native species are not adapted (the novel weapons hypothesis). Invasion success can also be related to increased resource availability, disturbances, or the presence of empty niches [121]. The diversity of these hypotheses (e.g. Catford et al. [121] describe 29 different ones) highlights the complexity involved and perhaps bodes caution when trying to link interactions of vectors with a group of organisms as broadly defined as “invasive alien plants”. However, many of these hypotheses do relate at least in part to biotic traits of the invasive organism. Further, invasives are often identified as such by their ecological or economic impact. To the extent that such broader ecosystem impacts are comparable among different invasive species, these characteristics may apply to mosquitoes as well. The questions of interest are then, which traits and impacts are well supported, and how do those traits intersect with the manner in which mosquitoes rely on local plant communities?

A number of such traits that could affect mosquitoes have been identified through large-scale comparative studies. These have shown that for a variety of traits related to plant physiology, including allocation of resources, growth rate, size, and fitness, invasive plants had greater trait values than native species [128]. Traits linked to invasiveness include a fast growth rate and vigorous spatial growth, particularly for IAP species in tropical areas [128], as well as greater photosynthetic rate and efficiency of water and N and P usage [129]. Potentially more salient findings, with regard to mosquitoes, are that IAP species have been found to be taller in their native range [130–132] (potentially affecting shading of habitats), and they have a different flowering phenology, either flowering longer than native plants [132–136] or tending to flower earlier or later [130, 137]. The majority of these studies were undertaken in temperate or Mediterranean ecosystems and therefore must be extrapolated with care to Afro-tropical regions. Such extended or earlier flowering periods, could, if associated with an extended or earlier production of nectar, enhance mosquito survival and population growth during periods when native plant communities might not support mosquito reproductive success.

A body of work also exists on ecosystem impacts associated with invasive species. For instance, water usage of plants, considered at an ecosystem scale (rather than at the level of an individual plant or even leaf), when evaluated within each growth form (grass/sedge, forb/fern, or tree/shrub), was found not to differ significantly between native and invasive plants. But a comparison among all growth forms pooled together showed that on average ecosystems dominated by IAPs had a 50% higher rate of evapotranspiration than those dominated by native plants [138]. Whether this might affect the microclimates of mosquito immatures (e.g. abundance of standing water) or of adults (e.g. humidity) is an open question. Further, IAPs have been found to decrease local plant abundance and species richness, but increase total community productivity (as measured in plant biomass or net primary productivity) [27]. The fitness as well as the abundance of animals also was lower, the latter by approximately 17% [27]. Likewise, an increase in biomass and reduction in plant diversity, causing a reduction in vertebrate animal abundance, may affect mosquito populations as well. This can cause a shift in host utilization of generalist blood-feeders such as *An. arabiensis,* resulting in a higher biting rate on humans. For instance, many IAPs are toxic and reduce pasture carrying capacities [23–25]. If this leads to a severe reduction in cattle near human habitations, this would result in higher biting rates on humans, due to reduced availability of domestic animals or to a genetic shift to a greater preference for human hosts. Malaria rates could be further exacerbated by any economic impacts of a loss of pasture. If invasive plants also affect non-domestic animals that do not serve as blood sources, including predators, competitors, pollinators, and honeydew producers, outcomes for the mosquito population become difficult to predict. Invaded ecosystems do tend to have a depauperate insect fauna, including fewer parasitoids and predators, and fewer phytophagous insects [139–145], which can result in a reduction in insectivorous birds [146–149]. It seems plausible, then, that such shifts in invertebrate diversity and abundance could favour mosquitoes, but this remains to be confirmed.

Evidence is scant that IAPs differ from non-invasive species in ways related directly to the fitness or vectorial capacity of vectors, and only a few studies have tested this explicitly. Traits having a direct effect would most likely relate to harbourages and to nectar, both of which can increase survival of adults. Features of favourable daytime harbourages may include plants that provide greater protection from wind, low humidity, direct sunlight, and excessive heat. Features of nectar that favour mosquitoes are its quantity and concentration, and the ease with which it can be accessed. The amount of accessible nectar is determined by physical characteristics of the inflorescence, interactions with invertebrates such as spiders or ants, and competition with pollinators, parasitoids, or other nectar feeders. Such tritrophic interactions have received little attention to date, and outcomes could conceivably go either way. For instance, if IAPs tend to escape from pathogens and herbivores, they may have less of a need to invest in the production of extra-floral nectar to encourage ants. On the other hand, if invasive plants are able to invest more energy into reproductive output, for instance by flowering and producing nectar for a longer period, and invertebrate communities tend to be diminished, this could result in a greater availability of nectar for mosquitoes.

**Fig. 1:**
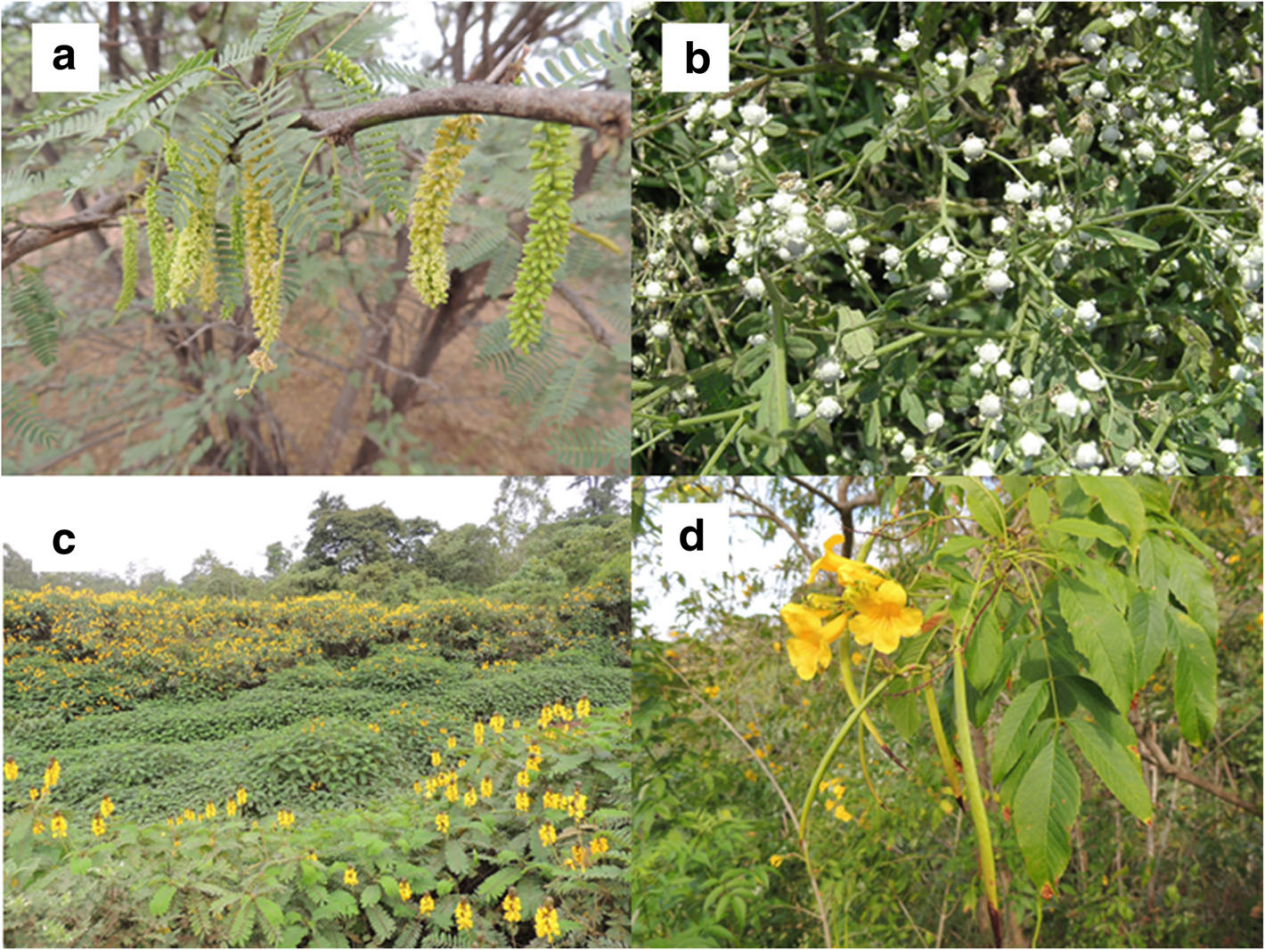
Invasive plant species in Africa known to be attractive to malaria vectors include *Prosopis juliflora* (**a**), *Parthenium hysterophorus* (**b**), *Senna didymobotrya* (**c**), and *Tecoma stans* (**d**)

A number of studies have assessed the survival of *An. gambiae* when provided with access to a variety of native and alien plants (though typically that distinction was accidental). In those studies, the plants that allowed the greatest longevity were those invasive in parts of Africa such as *Ricinus communis* L. (Euphorbiaceae) [51], *Manihot esculenta* Crantz (Euphorbiaceae) [62], and *Tecoma stans* (L.) Juss ex Kunth (Bignoniaceae) [54] (Fig. 1). Other IAPs however, such as *Lantana camara*, appear to provide only very little nectar and support longevities of only a few days [50, 51, 54, 150]. The same appears true of the weed *Parthenium hysterophorus* L. (Asteraceae) [54] (Fig. 1), although in one study this weed provided ample sugar and supported mean lifespans much closer to that of *R. communis* [151]. Whether such discrepancies are due to differences in experimental set-up (e.g. the condition of plants or cuttings that were used) or underlying genetic or environmental differences between plant populations, is an important open question. If there are extremely large differences in nectar production between populations of the same species of plant, recommendations for management will become that much more complicated.

Equally important is whether mosquitoes will locate and attempt to feed on nectar from non-native plants. A priori, one might assume that given the importance of nectar-feeding for mosquito reproductive success, mosquitoes will have evolved to respond most strongly to the volatiles of nectariferous plants native to their region. An alternative hypothesis is that the volatile organic compound blends released by plants provide a clue to their nectar productivity, and mosquitoes are able to detect such state-dependent cues. There is some support for the latter notion. For instance, when given access to a panel of plants, those that the mosquitoes perched on most often (i.e. were attracted to, or retained on the longest, in a cage) were also the plants that resulted in the greatest proportion of sugar-fed mosquitoes after exposure in a no-choice test, and these included a mix of native and alien plants [31]. Olfactometer experiments likewise have shown that both native [e.g. *Senna didymobotrya* (Fresen) H.S. Irwin & Barneby; Fabaceae,] and alien (*P. hysterophorus*) species to tropical Africa to be among the most attractive plants to *An. gambiae* (Fig. 1). In Mali, the most attractive flowering plants were *Acacia macrostachya* DC (Fabaceae), *Faidherbia albida* (Delile) A.Chev. (Fabaceae), *Boscia angustifolia* A. Rich (Capparaceae) and *Ziziphus jujube* Mill. (Rhamnaceae) [32], only the latter of which is an alien species and can become invasive in certain regions. In Burkina Faso, the most attractive plants were reported to include *Mangifera indica* L. (Anacardiaceae), *Delonix regia* (Hook.) Raf. (Fabaceae), *Thevetia neriifolia*, *Senna siamea* (Lam.) H.S. Irwin & Barneby (Fabaceae) and *Cassia sieberiania* DC (Fabaceae) [52], of which only the last is native to equatorial mainland Africa. This is further supported by a recent study finding that *An. gambiae* benefitted from the presence of the introduced invasive shrub *Prosopis juliflora* (Sw.) DC (Fabaceae) in Mali [35] (Fig. 1). Thus, there is little support for the notion that mosquitoes would favour plants with which they have co-occurred for longer (evolutionary) periods. Attraction studies that account for the state or condition of various plants could shed more light on this issue.

Finally, very little is known of the importance of biomass or abundance of different plants on the foraging decisions made by mosquitoes. Presumably (given that preferences for different plant species are not absolute), the plants that are fed on in a given locale will be a function both of their attractiveness or acceptability to mosquitoes, and of their abundance or how frequently plant species are encountered. This is particularly important for malaria mosquitoes such as *An. gambiae*, because sugar feeding and blood feeding are, to an extent, energetically interchangeable in this species. Thus, changes in plant abundance or biomass might affect not only which plants are used, but the rate at which humans are bitten.

To summarize, while traits and ecosystem impacts of IAPs appear highly variable and context dependent, those with the broadest support appear to be longer flowering durations, more vigorous growth (which may result in more flowers), and potentially an increase in biomass that provides more daytime shelter, sometimes in areas where previously little vegetation existed. Additionally, many IAPs may provide mosquitoes with ample nectar, increasing their longevity in the field. While one might expect mosquitoes to favour plants with which they have interacted over evolutionary time, alien plants are often among the most attractive plant hosts. So although many questions remain, it is at least plausible that invasive plants will increase malaria transmission rates.

## The potential for invasive alien plants to be managed on a large scale

### Invasive alien plant management

IAP management strategies need to include activities related to prevention, to early detection and rapid response, and to control. As most IAP species were intentionally introduced, the most effective way to preclude introductions into other regions is to prevent them. As such, a risk assessment of a plant’s potential for invasiveness should be undertaken prior to its introduction in these regions. Evaluated species that are deemed to pose a risk to natural resources, agriculture, or human and animal health should never be imported intentionally. The risk of unintentionally introducing invasive or potentially invasive species, especially in contaminated imports, can be reduced by imposing better controls at all ports of entry. If the authorities or designated officials have failed to prevent the introduction of an invasive or potentially invasive species, and it has established in the field, it is critical that it be detected early and eradicated, if possible, before it becomes widespread and abundant. To that end it is important that a surveillance strategy be developed and implemented so that small and isolated invasions can be contained and eradicated. If surveillance did not result in the early detection of a potentially problematic plant, and eradication is no longer feasible because it is already widespread and abundant, it is essential to implement a control strategy.

Control strategies can include the use of cultural, physical, chemical or biological methods or a combination of some or all of these measures, followed by rehabilitation or restoration. Cultural control (e.g. the use of fire, flooding or grazing, to reduce the abundance of invasive plants) can be effective on its own, or when used in conjunction with other control methods. Fire is especially effective for controlling succulents such as species in the Cactaceae and Crassulaceae, and can also be used to reduce the abundance of young seedlings or saplings of other IAP plants, even grasses. Total inundation of semi-aquatic plants by water, through controlled flooding, can also be used to manage semi-aquatic IAP species. Livestock, such as goats, are sometimes also used to control palatable terrestrial weeds, although results have been mixed - livestock can often contribute to the further spread of invasive plants.

Manual control involves the direct removal of the above-ground parts of a plant with an axe, saw, chainsaw or slasher, or the uprooting of plants using a hoe, garden fork or spade, or by hand pulling. Removal of the above-ground parts of a plant is suitable only for those weeds that do not coppice or regrow from the rootstock. Manual control may also include ring- and strip-barking of large shrubs or trees. Mechanical control may involve the use of heavy machinery or equipment (e.g. bulldozers or tractors and can, among others, involve pushing, stick-raking, blade-ploughing and/or chaining of larger plants or medium density infestations). There are a number of advantages of manual control in that practitioners require little training or supervision; tools are simple, cheap and easily obtainable. In most cases, little or no harm is caused to the environment and manual control can be used in countries where no herbicides are registered for use against a particular IAP species. However, there are disadvantages, as it often includes procedures that are labour intensive, and as such can be expensive in countries with high labour costs; it is physically demanding and slow, and it usually requires repeated follow-up operations; where machinery is used, manual control can be expensive - incurring fuel and maintenance costs; soil disturbance may stimulate seed germination among weeds, and on steep slopes or on riverbanks this may also exacerbate soil erosion; and in dense infestations, native species are often inadvertently damaged or removed.

Chemical control is the use of herbicides, applied alone or in combination with other methods and can be applied in several ways. Foliar spraying is the use of a diluted herbicide sprayed over the foliage (leaves and stems) of seedlings, shrubs, grasses or dense vine infestations to the ‘point of runoff’. Basal stem applications usually are applied to thin-barked woody weeds, tree saplings, regrowth and multi-stemmed shrubs and trees. The entire circumference of the trunk or stem from ground level to a height of 30–100 cm is sprayed or painted. Total frill involves the use of a hand-axe, panga, or machete, whereby horizontal cuts into the sapwood tissue of the stems or trunks of trees, vines or woody weeds, and then inserting herbicide into the cuts. Stem injection, sometimes also called drill-and-frill, involves the use of a battery-powered drill or similar tool. Holes are drilled (at a 45° downward angle) into the stems or trunks of trees, cacti, vines or woody weeds, and herbicides injected into the drill hole, using a squeeze bottle or plastic syringe. Stump applications involve the cutting down of a plant at the base of the stem and then immediately applying herbicide to the stump. Cut stump, sometimes also referred to as “cut and spray” or “lopping/pruning” involves the felling of a plant completely at its base (no higher than 15 cm above the ground), preferably horizontally by chainsaw, brush-cutter, or similar tools and then applying herbicide (with a paint brush, a squeeze bottle, a sponge-tipped bottle or a spray bottle) to the cut stump. Scrape and paint involves scraping a very thin layer of bark from a 10–30 cm section of stem (taking care not to cut through the vine), and then applying the herbicide to the exposed green underlying soft tissue before the plant can seal.

The main advantage of chemical control is that it is more cost-effective than other methods, especially manual control. Other advantages include the fact that results are quicker than with manual control, especially when compared with ring-barking or stripping, and that use of the correct herbicides, applied according to label recommendations, can have little to no negative impacts on the environment. However, there are also disadvantages, including the purchase of specialized equipment and the training of applicators, which can add to costs; herbicides can be expensive, especially if incorrect formulations are used, resulting in poor control and requiring repeated applications; target species must be ‘healthy’, and weather conditions suitable, at the time of a herbicide’s application; foliar applications can affect non-target species; herbicide misuse may cause environmental damage; and manual control of plants may be necessary before herbicide application. Also, widespread misuse of herbicides can produce severe environmental and health impacts [152, 153].

Biological control, that is, the use of host-specific natural enemies (pathogens, mites, and insects) to control invasive plants, has been practiced for many decades by a host of countries, especially the USA, Australia, South Africa, Canada and New Zealand. Over a period of 150 years, until the end of 1996, more than 350 species of invertebrates and pathogens were deliberately released in 75 countries for the control of at least 133 weed species [154]. It was estimated [155] that by the end of 2012, there were 1555 separate and intentional releases of 469 species of invasive-plant biological control agents against 175 species of invasive plants (when related taxa of unidentified plant species, such as some *Opuntia* spp. (Cactaceae), are counted as single target plants). These so-called ‘classical’ biocontrol projects have been conducted in a total of 90 countries [156]. At the national level, biocontrol programmes have achieved success rates of 83%, 80%, 61%, 51% and 50%, respectively, in New Zealand [156], Mauritius [157], South Africa [158], Australia [159] and Hawaii [160]. Analyses undertaken in South Africa more than 20 years ago revealed that six invasive alien plants out of 23 targeted were under complete control, and a further 13 under substantial control [161]. In Hawaii, more than 25 years ago, seven introduced weeds out of 21 were already under complete control, and substantial control had been achieved for three more [160]. In Australia, of 15 completed programmes, 12 resulted in complete control [159]. In South Africa, without biological control, the area occupied by the invasive cactus *Opuntia aurantiaca* Lindl. (Cactaceae) could have been 15 times greater than it is today [158]. Thanks to biological control *Opuntia ficus-indica* (L.) Mill (Cactaceae) invasions in South Africa have been reduced by approximately 90% [162, 163]. In fact the introduction of host specific and damaging agents has probably contributed 75% to the control of species in the family Cactaceae [164].

Some examples of biological control programmes against IAPs that have achieved some degree of control are given in Table 1. In general, programmes against aquatic species have, to date, achieved a high level of success.

**Table 1:** Examples of biological control programmes against aquatic and terrestrial invasive alien plants

Plant species	Family	Country/Region of control	Reference
Aquatic invasive plant target			
*Pistia stratiodes* L.	Araceae	USA; Africa	[165, 166]
*Azolla filiculoides* Lamarck	Salviniaceae	South Africa; Europe	[167, 168]
*Salvinia molesta* D. Mitch	Salviniaceae	Global	[169]
*Myriophyllum aquaticum* (Vell.) Verdc.	Haloragaceae	South Africa	[166, 170]
*Alternanthera philoxeroides* Griseb.	Amaranthaceae	Australia; New Zealand; Thailand; USA	[171]
Terrestrial invasive plant target			
*Prosopis* spp.	Fabaceae	Australia	[172, 173]
*Acacia* spp.	Fabaceae	South Africa	[174]
*Parthenium hysterophorus* L.	Asteraceae	Australia	[175]
*Ageratina adenophora* (Spreng.) R.M. King & H. Rob	Asteraceae	USA (Hawaii)	[159]
*Chromolaena odorata* (L.) R.M. King & H. Rob.	Asteraceae	Ghana; Indonesia; Marianas; USA (Guam)	[159]

The main aim of biological control is to suppress plant vigour, reduce seed production, slow plant growth, and reduce the density of the plant infestations. The main benefits of biological control according to Greathead [176] are these: agents establish self-perpetuating populations, often throughout the range of a target weed, including areas that are not accessible using chemical or mechanical control methods; the control is permanent; there are no negative impacts on the environment; the cost of biological control programmes is low, relative to other approaches, and requires only a one-off investment; benefits can be reaped by many stakeholders, irrespective of their financial status or contribution to the initial research process. Biological control also can be used to resolve “conflicts”. Many woody invasive species are widely promoted and disseminated for fuel wood in developing countries but also have negative impacts on livelihoods. Host-specific and damaging agents that attack only the reproductive parts of the plant can reduce spread and densification without reducing the beneficial attributes of the target species and in this way resolve the “conflict”. However, it should be noted that biological control agents are not available for every IAP species; some released agents have had negligible impacts on the target species; and there are situations where an agent has a significant impact on the target weed only in a small part of its adventive range. This is why control requires an integrated strategy where various options are used in combination in order to enhance suppression or elimination.

An issue that has not been addressed adequately is the cost-effectiveness of various control options. This need has led to a number of recent studies, mainly undertaken in South Africa, to determine if the benefits of invasive plant control outweigh the costs. For example, under a dynamic simulation of an ecological-economic model of IAP control in a mountain fynbos ecosystem in South Africa, it was found that the cost of proactive clearing would range from 0.6% to 4.76% of the economic value of ecosystem services, while resulting in an increase of the value of these services between 138 and 149% [177]. Also in South Africa, De Lange & van Wilgen [164] estimated the value of ecosystem services at ZAR 152 billion (presently, about US$ 11.551 billion) annually, of which an estimated ZAR 6.5 billion (US$ 490 million) is lost every year due to IAPs. However, the loss would have been an estimated additional ZAR 41.7 billion (US$3169 million) had no invasive plant control been carried out. Costs of aquatic weed control in Florida in the late 1960s were estimated to be US$ 6 million annually and benefits were reported as US$ 82 million, with the largest benefits coming from increased land use (due to drainage) and prevented flood damage [178].

Studies on the benefits of targeting individual species also have provided evidence of cost effectiveness. An analysis of the costs and benefits of the invasive Australian tree *Acacia mearnsii* De Wild. (Fabaceae) in South Africa suggests that a ‘do nothing’ scenario (with no attempts to control the spread of the species beyond the limits of commercial plantations) is not cost-effective, as the benefit: cost ratio is around 0.4 [179]. The most attractive control option will be a combination of biological control of the whole plant (flowers, seed pods, leaves and stems) and physical clearing (benefit: cost ratio of 7.5) [179]. Brown & Daigneault [180] found that an integrated approach to the control of the invasive tree *Spathodea campanulata* Beauv. (Bignoniaceae) in Fiji derived monetized benefits of US$ 3.7 for each US$ 1 spent, even without explicitly considering biodiversity, culture, and other non-monetized benefits of control. It is estimated that tamarisk (*Tamarix* spp.; Tamaricaceae) invasions in the western United States cost about US$ 280–450 ha^−1^ [181]. Eradicating these invasive species and restoring native riparian communities throughout the region would cost about US$ 7400 ha^−1^, considerably more than the current costs of impacts. However, these intervention costs would be fully recovered in as few as 17 years, after which the societal, ecological, and economic benefits of restoration would continue to accrue indefinitely [182].

Although there have been comparatively few studies to evaluate the overall costs and benefits of an integrated control strategy, the benefits of classical biological control programmes have been well documented. An analysis of some biocontrol research programmes in South Africa found that benefit: cost ratios ranged from 34:1 for *Lantana camara* L. (Verbenaceae) to 4331:1 for golden wattle, *Acacia pycnantha* Benth. (Fabaceae) [183]. In fact, the benefit: cost ratios for biocontrol projects in South Africa range from 50:1 for invasive sub-tropical shrubs to 3726:1 for invasive Australian trees (de Lange and van Wilgen, 2010 [164]). It is also estimated that biological control agents present in South Africa have reduced the financial costs of mechanical and chemical control by more than 19.8%, or ZAR 1.38 billion (presently, about US$ 104.8 million) [184]. It is further estimated that biological control programmes, if fully implemented in the future, may reduce control costs by an additional 41.4%, or ZAR 2.89 billion (presently, about US$ 219.5 million) [184]. These findings are supported by studies in Australia, which have found that every dollar invested in the invasive plant biological control effort yielded a return of A$ 23.10 [185]. There, the benefit: cost ratio for agriculture alone (in terms of both cost savings on control and increased production) was 17.4. If current annual expenditures on biological control research continue into the future, it is expected that projects targeting invasive plants with biological control agents in Australia may provide, on average, an annual net benefit of A$ 95.3 million, of which A$ 71.8 million is expected to flow into the agriculture sector [185]. A good example of the benefits of biological control is that of water hyacinth in southern Benin, where the reduction of this aquatic plant by biological control has been credited with an increase in income of US$ 30.5 million per year to a community of about 200,000 people [186]. If one assumes that the benefits stay constant over the next 20 years, the accumulated present value will be US$ 260 million - a benefit: cost ratio of 124:1 [186].

In summary, the most cost-effective way of controlling invasive plants is by combining two or more of the methods mentioned above. For example, manual control used in combination with chemical and/or biological control, commonly known as integrated pest management (IPM), should be implemented wherever possible in order to reduce costs and improve the efficacy of control across a landscape. Invasive plants can be effectively controlled over large areas by developing and implementing an integrated management strategy.

## The potential effect of invasive alien plant management on the incidence of malaria

If in certain regions IAPs are exacerbating malaria transmission, it would be useful to know what will occur if the distribution and densities of these weeds are reduced. For certain scenarios this appears straightforward. If the invasion of alien plants leads to increased poverty by reducing crop yields and pasture productivity, then improving people’s socio-economic conditions should, to an extent, help them escape the poverty trap of malaria by providing increased access to medication and preventive measures. In arid areas where an IAP might have expanded the range of a malaria vector, removal of the weed would likely reverse this expansion [35]. A similar argument would hold for cases where an invasive plant might prolong the seasonal population peaks of vectors. Support for the latter notion comes from a recent field trial in Mali, where the impact of the invasive shrub *Prosopis juliflora* on malaria mosquitoes was investigated during the dry season [35]. Villages without the plant were compared to those where *P. juliflora* had become established. In half of the latter villages, at a certain point all flowering branches of this plant were removed. *Anopheles* spp. were monitored throughout, to examine whether removal of this invasive putative nectar source affected mosquito species composition, age structure, population size, and sugar-feeding status. The average number of female *Anopheles* caught per trap declined more than two-fold in the villages where inflorescences had been removed, while numbers stayed stable in the positive and negative control villages. Likewise, the proportion of females that survived for at least 3 gonotrophic cycles dropped from 35% to 11%. Sugar-feeding rates also dropped dramatically following removal of the flowering branches. These results are similar to those of a study that indicated that in areas with more abundant or richer nectar sources, *Anopheles* spp., mosquitoes would live longer and greater populations would be sustained [64]. Another notable result was that the species composition shifted from a mix of *An. coluzzi*, *An. gambiae* and *An. arabiensis* to one dominated by *An. coluzzi*. Whether this species shift toward *An. coluzzi* reflects its lower dependence on nectar, or perhaps a tendency to make use of other sources available in arid environments (for instance, by piercing plant tissues), remains to be investigated. It also will be important to investigate the impacts of (i) invasive plants on mosquitoes throughout the year, not just in the dry season; (ii) other IAP species (both terrestrial and aquatic), to determine whether these effects are mosquito-plant species-specific or instead applicable to invasive species and mosquitoes in general; and (iii) invasive plants in a wider range of habitats, particularly in more verdant areas where mosquito-plant interactions will be far more complex. Another recent invasive plant-removal experiment performed in North America suggests that at least some of these aspects may be common. In a 2-year study using a Before-After/Control-Impact design, [187] found that the abundance of *Culex* spp. declined following removal of invasive Amur honeysuckle (*Lonicera maacki*). Although they did not measure vector survival rates directly, they did find a more favourable microclimate for mosquitoes in areas with honeysuckle. Neither study evaluated the impact on pathogen transmission, but it could be considerable. A quick calculation shows that in the case of *P. juliflora* inflorescence removal, assuming that only vector mortality and abundance would change by the levels indicated in the paper [35], and assuming a gonotrophic cycle length of 3 days and an extrinsic incubation period of 12 days, *R*_*0*_ would be reduced by a factor of approximately 28. However, as reviewed above, many of the other behavioural and life history traits of mosquitoes also may change with changes in vegetation, and it will be important to study such impacts in a comprehensive manner in future experiments.

Less clear is what happens if an invasive plant merely replaces part of the local plant community and (perhaps) increases the available plant biomass and nectar in that region. There are several important questions that cannot in an obvious way be extrapolated from the laboratory and semi-field experiments that have been done to date. The initial questions are these: Which plants tend to be replaced in invaded landscapes, those that already provide nectar to mosquitoes, or those that are poor nectar-hosts or relatively unattractive to mosquitoes? And by how much does the availability of nectar change in the landscape, whether as a result of different nectar production rates, or changes in the invertebrate community within the landscape? If there is generally an increase in the availability of nectar, how does this affect mosquito nectar-feeding, human-biting behaviour, reproductive success, and population size throughout the year? It is worth noting that in most studies there were only two levels of nectar abundance, high and low, or present and absent. To understand how invasive plants might affect vectorial capacity, we would require insights into mosquito traits as a function of nectar availability. For instance, it is likely that there is a lower limit of nectar availability at which mosquito populations can be sustained [73], but whether that lower level is ever relevant in nature, i.e. whether nectar ever becomes limiting in nature, remains unknown [30]. Likewise, above a certain level of nectar abundance, further increases may have limited impact on mosquitoes. An additional complication is that nectar feeding appears to be more relevant to energetically-deprived or smaller mosquitoes, and when blood hosts are inaccessible or absent [68, 188]. It is possible that this interaction with blood-host accessibility or presence explains why in some circumstances where sugar access is greater, the biting rate and resulting vectorial capacity of the mosquito population is lower [50, 66]. Thus, ideally we would need to measure mosquito traits as a function of nectar abundance at different levels of blood-host presence.

In practical terms, another point of uncertainty relates to the intensity of transmission that occurs in a given region. In areas where vectorial capacity or *R*_0_ (or alternatively, the proportion of humans that are parasitemic) is very high, a strong reduction in vectorial capacity and therefore *R*_0_, may still have little impact on malaria prevalence. It is only in areas with a lower transmission pressure that reductions in vectorial capacity will have a more pronounced impact on malaria incidence. This suggests that if management of nectar sources of mosquitoes has a strong impact on vectorial capacity, it could potentially contribute in a significant way to malaria control in areas of low transmission. Alternatively, plant removal could be considered as one of many components of an integrated control strategy in high transmission areas. This might be particularly relevant in areas where use of long-lasting insecticidal nets and human-case management are insufficient to interrupt transmission, or areas where insecticide resistance is climbing.

The relationship between many *Anopheles* species and aquatic IAPs is often strong enough to warrant invasive plant control as an additional malaria management tool. The same can be said for many mosquito species and other disease vectors. We must ask ourselves if manipulating the environment to control any given malaria vector includes the risk of creating suitable environments for other malaria vectors or vectors of other diseases. The current evidence suggests this would not be the case: removal of emergent vegetation to control *An. funestus*, for instance, would not necessarily create more good habitats for *An. gambiae* (*s.l.*) and other vectors, inasmuch as large bodies of exposed, deep, clear water are unsuitable for oviposition and larval development of mosquitoes in general [75, 76, 78, 88–90]. Added to the economic and environmental benefits of applying biological control for aquatic weeds, it is apparent that malaria suppression also could profit from aquatic invasive plant management, which already has had successes.

## Conclusions

This review highlights the complexity of the *Anopheles*-plant relationship and the necessity of understanding it, in order to anticipate how and when IAPs may increase malaria incidence. By using our knowledge of the interplay of factors influencing this relationship from the pathogen’s perspective, it appears we can judiciously apply invasive-plant interventions to suppress malaria transmission, or even to interrupt it altogether in some instances. Field experiments focused on unknown features of the mosquito-plant interface will yield more information needed to know best how to approach the invasive-plant problem. Initial investigations should use the entomological inoculation rate (EIR) to compare malaria exposure in areas with similar housing conditions, human density, socioeconomics, and bed-net usage, leaving only alien-plant establishment as the variable. If the comparison indicates a strong impact of these plants on *Plasmodium* exposure, further studies on mosquito foraging behaviour and its implications for population dynamics and vectorial capacity will be revealing and provide further insights. These will inform how IAP management can contribute to malaria control and ensure that programmes targeting different aspects of environmental and human health are to be coordinated in a beneficial manner.

## Abbreviations

*C*: Vectorial capacity
EIR: Entomological inoculation rate
*N*_*h*_: Population size of humans
*N*_*v*_: Population size of vectors
*R*_0_: Basic reproduction number of malaria
ZAR: South African rand
